# Student Paramedic and Practice Educator Experiences of Ambulance Placements. A Rapid Evidence Review

**DOI:** 10.1002/hsr2.70552

**Published:** 2025-03-18

**Authors:** Joseph James Hudson Brown, Marishona Ortega, Gregory Adam Whitley

**Affiliations:** ^1^ School of Health and Care Science University of Lincoln Lincoln UK; ^2^ Libraries and Learning Skills University of Lincoln Lincoln UK; ^3^ Clinical Audit and Research Unit East Midlands Ambulance Service NHS Trust Lincoln UK

**Keywords:** education, emergency medical services, paramedics, students

## Abstract

**Background:**

Ambulance practice placements overseen by paramedic practice educators form an essential aspect of training for student paramedics; however, this approach, adapted from other healthcare professions, is a relatively new model for paramedicine and evidence regarding its application in prehospital contexts is limited. Therefore, this rapid evidence review aimed to explore student paramedic and paramedic practice educator experiences of ambulance practice placements.

**Methods:**

EBSCOhost was used to search the Cumulative Index to Nursing and Allied Health Literature Complete and MEDLINE databases from inception to March 2024. Screening and data extraction was performed by one reviewer and verified by the second. Included studies were appraised using the Critical Appraisal Skills Programme qualitative checklist and thematic synthesis of results undertaken.

**Results:**

Of 134 records screened, seven were included in the review, representing 131 participants from the United Kingdom, Australia and New Zealand. Four themes were identified, including practice educator training and support, student‐practice educator relationships, organization and communication, and operational factors.

**Conclusion:**

Outdated workplace practices, personal factors, education‐industry barriers and unpredictable working environments impacted student paramedic and paramedic practice educator experiences of ambulance practice placements. Better communication is needed between universities and ambulance services to improve the consistency of ambulance practice placements for student paramedics and paramedic practice educators.

## 1 Introduction

There are around 40,000 Health and Care Professions Council (HCPC) registered paramedics in the United Kingdom (UK) with the majority (around three‐quarters) employed by National Health Service ambulance service trusts [1, 2]. Historically, paramedics evolved from ambulance staff providing a municipal transport service into prehospital specialists associated with the delivery of advanced emergency care to patients with serious injury or illness [3, 4]. However, prehospital medicine has transformed over the last two decades in response to factors such as ageing populations, chronic ill health and rising demand on mental health services [5]. Therefore, as well as managing increasing volumes of emergency calls, pressure on ambulance services to deliver more complex and urgent care in a broader range of settings has increased [5, 6]. Consequently, the need for a step change in paramedic training and education was identified [7, 8]. Scopes of practice were expanded, clinical specialisms in primary, urgent and critical care developed and, in 2018, paramedicine became a graduate entry profession to standardize programs of study and maintain proficiency at the required depth [7, 8, 9, 10, 11, 12, 13]. Paramedics are now defined as autonomous generalist clinicians practicing across a range of emergency, primary or urgent care settings, who may also specialize in education, leadership and research [14, 15].

Student paramedics in the UK are either ambulance service emergency medical technicians enrolled on in‐service degree apprenticeship schemes, or external students who apply directly to university [16, 17]. Both follow HCPC approved programs of study evenly split between taught theory and a range of healthcare practice placements, the majority of which are ambulance based and organized in association with partner ambulance services [18, 19]. Students are mentored, taught, coached and assessed by paramedic practice educators (PEds), who form a vital link between universities and industry [20]; however, supervised prehospital practice education is relatively new in the context of paramedicine and remains under researched [21, 22]. This rapid evidence review aims to explore student and PEd experiences of participation in supervised ambulance practice placements to better understand how their organization and structure may impact teaching and learning and influence future training pathways for paramedics.

## 2 Methods

### 2.1 Study Design

Research is an intentionally planned investigation of a defined context using established methodologies to produce transferrable findings [23]; therefore, an inductive narrative review study design was used to answer the following question, developed from the PICo (population, phenomena of interest, context) framework [24]:

*What are student paramedic and paramedic practice educator (PEd) experiences of ambulance practice placements?*

Inductive narrative reviews provide an overall summary of a subject with elements of interpretation and analysis and derive themes from the data itself [25, 26, 27]. They usually rank below systematic reviews in hierarchies of research evidence [28, 29]; however, arguably provide a wider perspective and deepen understanding [30], which supports the use of an inductive narrative review approach in this study. For the purposes of this review student paramedics were defined as learners enrolled on a higher education course leading to professional registration as a paramedic. PEds were defined as qualified paramedics with responsibility for ensuring clinical supervision, leadership and development of a student paramedic [20]. Ambulance practice placements were defined as ambulance based clinical or practical experience forming part of an approved program [31].

### 2.2 Search Strategy

On 01 March 2024 EBSCOhost was used to search Cumulative Index to Nursing and Allied Health Literature Complete and MEDLINE from inception to March 2024 with the following terms and Boolean operators:

(paramedicine* OR prehospital OR ambulance OR paramedic OR “emergency medical service*”) AND (preceptors OR supervisor OR instructor OR “practice educator” OR mentor) AND student

Only original research published in English language peer reviewed academic journals was included, which ensured only pertinent, higher‐quality evidence in a language that could be understood were retrieved. Additional studies for inclusion were identified via reference list checking and internet searches. Duplicates were removed before screening. The search strategy was informed by an academic librarian (MO).

### 2.3 Study Screening and Data Extraction

Search results were exported into Microsoft Excel [32], title and abstract screening was performed by JJHB, and verified by GAW. Full text articles were sourced and screened against inclusion and exclusion criteria by JJHB and verified by GAW. Extracted data included author, date of publication, title, research method, study population, focus of interest, context, and outcome. This was performed by JJHB.

### 2.4 Critical Appraisal Tool

As all included studies were qualitative in design their quality and validity were assessed using the Critical Appraisal Skills Programme qualitative checklist [33], performed by JJHB and verified by GAW.

### 2.5 Synthesis Technique

Thematic synthesis refers to the systematic coding of data to generate descriptive and analytical themes [34]. A thematic synthesis of extracted data was undertaken by JJHB to identify recurring themes relevant to the research question. Included articles were analysed, common themes highlighted, and findings tabulated. Data were then condensed into units of meaning (sentences) and grouped according to theme.

## 3 Results

### 3.1 Study Screening

**Figure 1:**
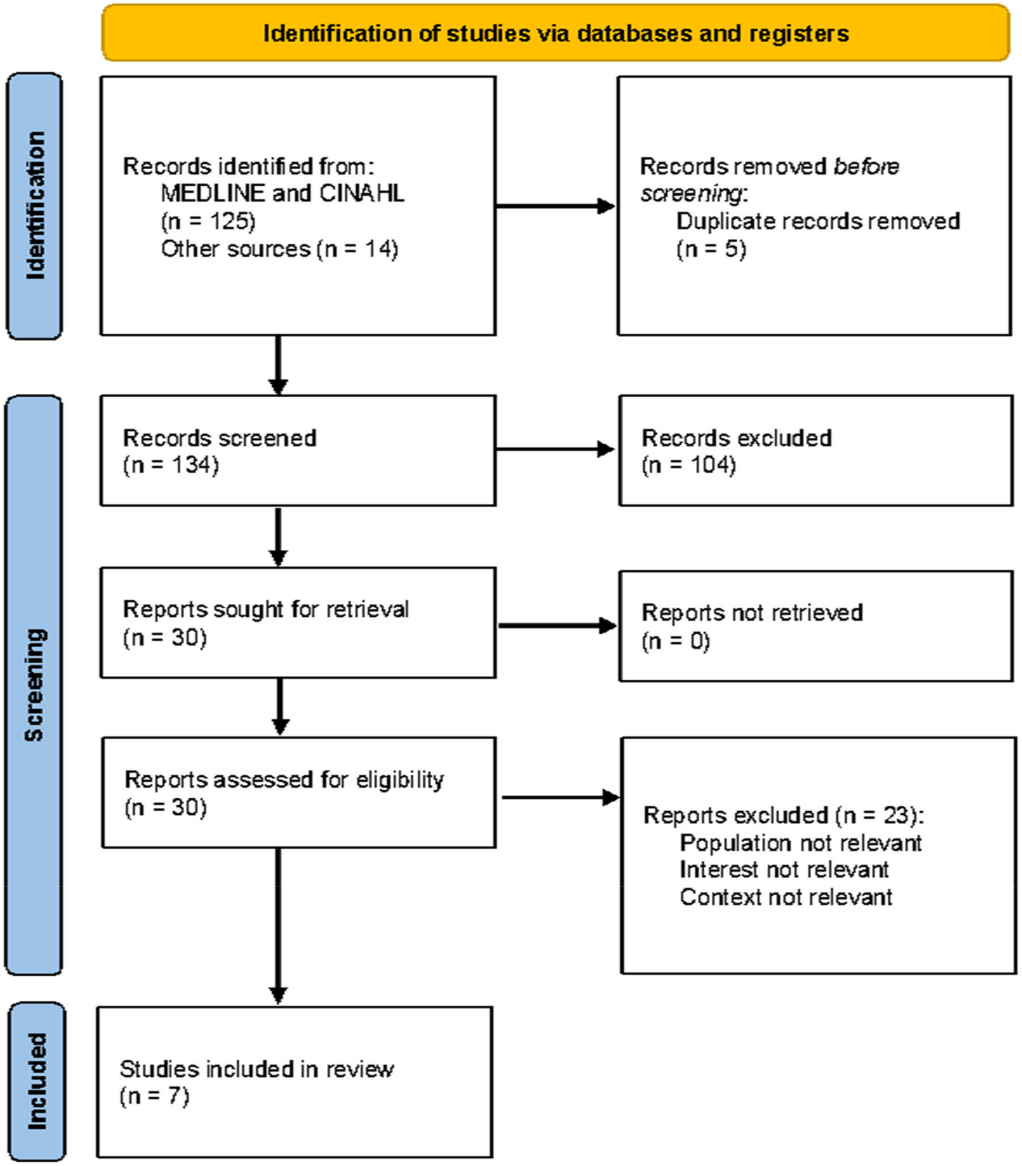
PRISMA flow diagram [35].

139 records were retrieved using the described search strategy. Five duplicate records were removed. 134 records were screened by title and abstract and 104 records not relevant to the research question excluded. Full text PDFs of the remaining 30 studies were downloaded and read, with 23 excluded as not relevant to the research question population (student paramedics or paramedic PEds), phenomena of interest (experiences) or context (ambulance practice placement). This left seven studies included in the review, all original research published in English language peer reviewed academic journals. See Preferred Reporting Items for Systematic Reviews and Meta‐Analyzes (PRISMA) diagram [35] (Figure 1).

### 3.2 Summary of Included Studies

131 participants across seven qualitative studies conducted between 2014 and 2024 were included. All studies explored ambulance practice placements: two studies focused on student experiences, three on PEd experiences and two on both student and PEd experiences. Four studies explored ambulance practice placements in the UK (including one from Scotland), two studies placements in Australia and two studies placements in Australia and New Zealand. These data provided the basis for synthesis (Table 1).

**Table 1:** Summary of included studies.

Author/date/title	Method	Population	Interest	Context	Outcome
Lane [36]**:** Student perceptions in relation to Paramedic Educator (PEd) roles	Framework analysis of qualitative data generated via semistructured individual face‐to‐face interviews.	Final year paramedic students from three universities(*n* = *8*).	Student perceptions in relation to paramedic PEd roles.	UK ambulance practice placements.	Mentoring culture within UK ambulance services insufficiently established to support students and PEds.
O'Meara [37]**:** Starting the conversation: What are the issues for paramedic student clinical education?	Thematic analysis of qualitative data generated through a community conversation approach.	Students, PEds and ambulance service management attending a paramedic conference (*n* = *53*).	Issues affecting paramedic student clinical education and possible solutions.	Australian ambulance practice placements.	Ambulance placement planning/organization requires improved communication between universities, ambulance service, PEds and students.
O'Meara [38]**:** Paramedic instructor perspectives on the quality of clinical and field placements for university educated paramedicine students	Thematic analysis of qualitative data generated via semistructured individual face‐to‐face interviews.	Experienced paramedic PEds (*n* = *15*).	PEds perspectives on the quality of clinical placements for paramedic students.	Ambulance practice placements in Australia and New Zealand.	Lack of agreed standards for quality practice placements. Vocationally trained PEds valued paramedic practice placements most, while university educated PEds also valued placements in more diverse settings.
Lane [39]**:** Mentorship within the paramedic profession: a practice educator's perspective	Thematic analysis of qualitative data generated via semi‐structured face‐to‐face focus group interviews, informed by previous study [36].	Paramedic PEds from two ambulance services (*n* = 14).	PEds' perspective of mentoring within paramedic practice.	UK ambulance practice placements.	PEds desire greater investment by ambulance services/universities to tackle organizational barriers to effective mentoring.
Baranowski [40]**:** Do non‐rotational ambulance‐based placements affect the development of paramedic competencies from a student perspective? A qualitative study	Thematic analysis of qualitative data generated via semi‐structured face‐to‐face focus group interviews.	Second year undergraduate paramedic students from a single university (*n* = *9*).	Whether having the same paramedic PEd affects students' development of competencies.	UK ambulance practice placements.	Insight into how students perceive having the same PEd, but inconclusive as to their effect on development of competencies.
Smith [41]**:** Undergraduate paramedic student competency assessment: A grounded theory study explaining how assessors in Australia and New Zealand determine student competency to practice	Glaserian Grounded Theory analysis of semistructured face‐to‐face individual (*n* = 2) and focus group (*n* = *5)* interviews.	Paramedic lecturers (*n* = 8) and PEds (*n* = 14).	How PEds determine the clinical competency of paramedic students on practice placements.	Ambulance practice placements in Australia and New Zealand.	Developed the Paramedic Assessment Process (PAP) theory where *engaging*, *measuring* and *moderating* linked with *aligning* explain assessment process.
Worsfold [21] “This is how I'm going to do it, but this is not how you're going to do it”: the expectation gap between student paramedics and mentors in East and Central Scotland	Thematic analysis of qualitative data generated via semistructured online focus group interviews.	Scottish paramedic students from a single university (*n* = *8*) and experienced PEds (*n* = *2*).	Practice placement expectations gap between students and PEds.	Scottish Ambulance Service practice placements.	Identified increased need for training and support for PEds, alongside stronger links between university and Scottish Ambulance Service.

### 3.3 Critical Appraisal Results

Critical appraisal is the systematic evaluation of research to judge its validity, trustworthiness and relevance in a particular context [42]. Critical appraisal of studies included in this review found: two were unclear regarding participant recruitment strategy; three did not clearly describe the relationship between research and participants; two gave unclear consideration to ethics; three lacked clarity regarding the rigor of their data analysis; and one was of unclear validity (Table 2). These factors limit the veracity of conclusions synthesized from the evidence.

**Table 2:** Critical appraisal results [33].

	Lane [36]	O'Meara [37]	O'Meara [38]	Lane [39]	Baranowski [40]	Smith [41]	Worsfold [21]
1. Was there a clear statement of the aims of the research?							
2. Is a qualitative methodology appropriate?							
3. Was the research design appropriate to address the aims of the research?							
4. Was the recruitment strategy appropriate to the aims of the research?							
5. Was the data collected in a way that addressed the research issue?							
6. Has the relationship between researcher and participants been adequately considered?							
7. Have ethical issues been taken into consideration?							
8. Was the data analysis sufficiently rigorous?							
9. Is there a clear statement of findings?							
10. How valuable is the research?							

**Key**	**Yes**	**Unclear**	**No**

### 3.4 Synthesis of Evidence

**Figure 2:**
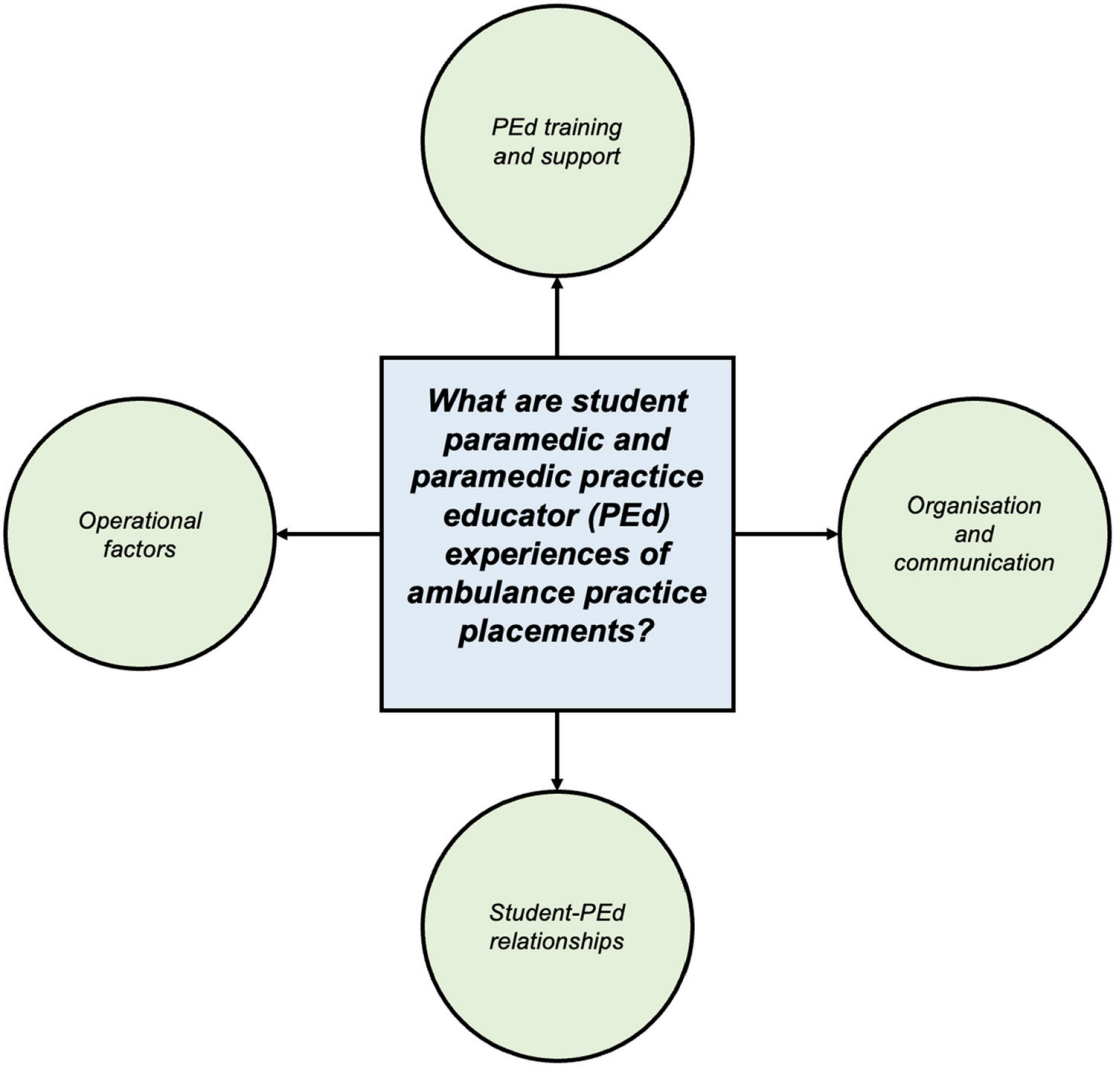
Synthesized themes.

Key themes synthesized from the evidence focused on PEd training and support, student‐PEd relationships, organization and communication and operational factors (Figure 2).

#### 3.4.1 PEd Training and Support

Students identified PEds with greater training and experience in higher education, good clinical knowledge and a thorough understanding of the paramedic role as better facilitators of positive learning environments [21, 36]. PEds with greater experience of academia were also positive toward degree level study, saw benefits in placement diversity and embraced evidenced based practice [38, 39]. However, PEds trained vocationally were skeptical regarding a graduate entry profession, seeing paramedicine as a practical job, wanting ambulance placements prioritized [21, 38] and citing a theory‐practice gap between knowledge disseminated to students in university and PEd experience [21, 38, 41]:

I think there are people that just do not want to put in the effort… a lot of staff have been in the job for several years and argue that well, it's not an academic job

[Practice educator] [21]

Students recognized the benefits of effective mentoring and role modeling but expected better training and support for PEds [21, 36].

Likewise, PEds recognized their role was rewarding, could benefit their knowledge and skills and improve staff retention; however, some found it stressful, not valued or adequately rewarded and that support was inconsistent [21, 36, 38, 41]:

It would be very nice to have a situation where the mentors have a mentor to mentor the mentoring

[Practice educator] [39]

Students believed paramedicine had evolved and that PEds should support theoretical as well as clinical development [21], further respecting PEds who acknowledged gaps in their own knowledge and engaged in mutual learning opportunities [36]. However, students also reported PEd resistance to this, which led to inconsistent teaching and assessment and lowered their placement expectations [21]. This was echoed by PEds, who described how unfamiliarity with higher education systems and inadequate training and support negatively impacted their understanding of students' developmental level, leading to unequal learning opportunities and inconsistent assessments [21, 39, 41].

#### 3.4.2 Student‐PEd Relationship

Students expressed a desire for friendship with PEds, framing this relationship through shared knowledge, experiences and values [21, 36, 40]; although they also recognized this made critical feedback harder to hear and impacted negatively on fair assessment [21, 36]:

You are spending 4 days together and it's in close proximity, you know. You got to have this relationship where you can talk about, not just work, but things outside of work as well

[Student paramedic] [36]

By contrast, PEds saw themselves in a parenting role and were cautious of befriending students due to conflicts of interest [21, 41]. They also described how workplace gossip and personal opinion led to inconsistencies in student assessment [41], and how they adjusted their placement expectations depending on students' previous life and work experiences [21]:

I think it all depends on their background… I think when you get the students who are younger or haven't really had that kind of life experience, your expectations change

[Practice educator] [21]

Students and PEds alike valued enthusiasm, motivation, kindness, humor and respect in one another [36, 39]. Students also linked consistent placement with the same PEd to improved learning opportunities and mental well‐being [36, 40]. They described how good PEds inducted them into wider paramedic culture, facilitated effective learning environments and were respectful; however, bad PEds created barriers to these features, were unkind and unfriendly and caused students to seek alternative arrangements [21, 36, 40]. Some students even reported altering their conduct to please PEds and fit in on placement, including adopting and tolerating inappropriate behavior and attitudes [36, 40]:

#### 3.4.3 Organization and Communication

Students and PEds reported poor organization, communication and tension between universities and partner ambulance services, which caused a lack of placement continuity and impacted negatively on learning environments:

Who decided that being a paramedic would involve a university route now… they throw you into ambulance service placements, and the ambulance service is like, “What?” Like, “we were not prepared for this”

[Student paramedic] [21]

Students discussed the detrimental effect this had on their finances and mental well‐being [36, 37, 40]. PEds, meanwhile, found it impeded accurate assessment, clarity regarding raising fitness to practice concerns and contributed to levels of work‐related stress [37, 38, 41], driven in part by increased pressure to sign off student competencies and the implications of this for patient safety and their professional credibility [37, 38, 41]:

On his mentoring report I recommended he not carry through… they (university) still put him through. So, what's the point?

[Practice educator] [41]

However, overall, students enjoyed placement and were positive about diversification of settings; although they voiced concerns regarding repetition of skills and a lack of clinical opportunities at rural stations [37, 40].

#### 3.4.4 Operational Factors

Students reported operational pressure on ambulance services resulted in fewer opportunities for welfare checks, structured feedback or simulated teaching and discussed feeling guilty for adding to the stress and workload of PEds [21, 36]. PEds also recognized the negative impact providing a time‐critical service while managing unpredictable and challenging situations had on the learning environment and their ability to consider the mental well‐being of students [39, 41]:

The difficulty with our profession is we're getting busier and busier, and the pressures get more and more, and things like welfare get put to the back of the pile

[Practice educator] [39]

Students cited random allocation of jobs as limiting clinical exposure [40]:

Where I am there's quite a lot of breathing difficulty jobs… so you seem to become good at one assessment type really… there's not too much varied in that sense so it can be a bit frustrating at times

[Student paramedic] [40]

They also highlighted working unexpectedly with new PEds due to rota changes disrupted continuity of teaching, created additional performance pressure, impacted learning environments, made assessment a tick box exercise and challenged their mental resilience by reducing opportunities for follow up welfare checks [36, 37, 40]. PEds reported a reluctance to engage with new theories or practice under such circumstances and were aware how this may further contribute to a theory‐practice gap [38, 41]; however, they regarded this a mitigating factor in the assessment of students, while recognizing students representing a risk to patient safety should not be passed as competent [41].

## 4 Discussion

This rapid evidence review aimed to explore student and PEd experiences of ambulance practice placements to better understand how their organization and structure may impact teaching and learning and influence future training pathways for paramedics.

We found training and support for PEds was described as variable by students and PEds, with PEds also citing a lack of recognition for their role. PEds reported fulfilling a multifaceted role similar to nurse mentors [43, 44]; however, unlike paramedicine, one‐to‐one nurse mentors in the UK have been replaced with a three‐to‐one tripartite model to improve support for educators, encourage collective responsibility and decision making and raise placement quality [45, 46, 47]. Langford et al. [48] explored transposing a similar nursing facilitator model to paramedic ambulance placements in Australia. They found university employed facilitators with responsibility for educational oversight working alongside PEds improved placement quality, created a more positive experience for students and PEds and bolstered education‐industry links. This is significant as we found students and PEds experienced inconsistent organization and communication between education and industry during practice placement, which increased stress and impacted negatively on systems of teaching, learning and assessment. Clarke [49] attributed this to a disconnect between theory (teaching) and practice (doing) caused, in part, by the transition of paramedic education from vocation‐based training to degree level study with professional registration; although more research is required. This suggests nursing facilitator models may improve training, support and recognition for PEds, strengthen organization and communication between education and industry and help moderate the theory‐practice gap. We also found student‐PEd relationships were described as a significant influence on students' experiences of placement as well as PEds' assessments of students, although students and PEds agreed on positive and negative personal qualities in one another. These findings are echoed in recent reviews of English ambulance service culture, which identified bullying, harassment (including sexual harassment) and inadequate support of speaking up as barriers to change [50, 51]. Whether this is indicative of systemic cultural issues internationally remains uncertain. Finally, we found operational factors and the unpredictability of prehospital practice were reported by students as creating barriers to clinical exposure during placement and impacted PEds ability to support students' clinical development and well‐being. Cimino and Braun [52] suggest health technologies, artificial intelligence, simulation, standardization of protocols and guidelines and better collaboration between healthcare providers may help to mitigate the unique challenges of prehospital practice and research and, in turn, help reduce the impact of unpredictability and operational factors on paramedic prehospital education.

### 4.1 Strengths and Limitations

This rapid evidence review was undertaken by a single researcher and is therefore at risk of significant bias; however, established systematic research and synthesis techniques were used to mitigate this. The search strategy only included two databases, with search terms generated through trial and error, and only studies published in English‐language academic journals were included; therefore, relevant studies may have been missed. Title and abstract of search records were systematically screened against clear inclusion and exclusion criteria; however, studies with irrelevant titles but relevant subsections may have been missed. Included studies were critically appraised using an externally standardized and validated tool to reduce bias, although alternative tools may have given a different result. Reviewed evidence was international, allowing evaluation of experiences across different cultures and ambulance services; however, included countries were all developed nations so review findings may not be reflected in the developing world where, as well as being underfunded and under resourced, emergency medical services are often fragmented across multiple public and private organizations with little integration or standardization of protocols [53]. Finally, this review included qualitative data alone, which can provide a deeper and more holistic understanding of a phenomenon [54]; however, the inclusion of quantitative data may have provided additional objectivity and generalizability [55].

### 4.2 Implications for Clinical Practice and Future Research

Within practice, partner ambulance services should enhance organization and communication with universities to improve placement consistency for students and PEds. They should also challenge outdated cultures by integrating new ways of working and enabling speaking up. Future research exploring how the move of paramedicine toward a graduate‐entry profession is impacting practice placements is warranted, for example comparing experiences of in‐service and direct‐entry students and how well prepared they feel for autonomous clinical practice, or exploring whether attitudes toward the PEd role vary between paramedics registered before 2018 and more recently qualified graduates. Trials of nursing facilitator models in paramedic practice placement settings are also justified, as well as continuing research into the application and impact of technology on prehospital healthcare and education. The findings of such research could have important implications for the future structure, organization and funding of paramedic training pathways. Further research regarding the prehospital application of healthcare educational assessment tools, such as the Undergraduate Clinical Environment Education Measure [56], may also help amplify the voice of student paramedics in shaping placements.

## 5 Conclusion

This narrative review explored student paramedic and paramedic PEd experiences of ambulance practice placements. The methodology identified four key themes from the evidence: PEd training and support; student‐PEd relationships; organization and communication; and operational factors. Outdated workplace practices, personal factors, education‐industry barriers and unpredictable working environments are all implicated in significantly impacting student paramedic and paramedic PEd experiences of ambulance practice placements. A need for better communication between universities and ambulance services and increased placement consistency for students and PEds was identified in the evidence.

## Author Contributions

**Joseph James Hudson Brown:** conceptualization, formal analysis, methodology, project administration, software, writing – original draft. **Marishona Ortega:** methodology, writing – review and editing. **Gregory Adam Whitley:** methodology, writing – review and editing, supervision.

## Conflicts of Interest

The authors declare no conflicts of interest.

## Transparency Statement

The lead author Joseph James Hudson Brown affirms that this manuscript is an honest, accurate, and transparent account of the study being reported; that no important aspects of the study have been omitted; and that any discrepancies from the study as planned (and, if relevant, registered) have been explained.

## Data Availability

The authors have nothing to report.
